# Use and retention of long-lasting insecticidal nets (LLINs) in a malaria risk area in the Brazilian Amazon: a 5-year follow-up intervention

**DOI:** 10.1186/s12936-019-2735-9

**Published:** 2019-03-25

**Authors:** Jessica Oliveira de Sousa, Bernardino Claudio de Albuquerque, José Rodrigues Coura, Martha Cecilia Suárez-Mutis

**Affiliations:** 1 Laboratory of Parasitic Diseases, Institute Oswaldo Cruz/Fiocruz, Av Brasil 4365. Pavilhão Artur Neiva, Rio de Janeiro, RJ CEP: 21040-900 Brazil; 2 Foundation of Health Surveillance of Amazonas, Av. Torquato Tapajós, 4.010, Colônia Santo Antônio, Manaus, AM CEP 69.093-018 Brazil

**Keywords:** Malaria, LLINs, Use, Retention, Control, Elimination

## Abstract

**Background:**

Long-lasting insecticidal nets (LLINs) are one of the main vector control strategies recommended by the World Health Organization for the control and elimination of malaria. This study aimed to evaluate the use and retention of LLINs during the 5 years of implementing an integrated control strategy in a malaria-endemic area in the Brazilian Amazon.

**Methods:**

This intervention study was conducted in localities of the municipality of Barcelos, Amazonas, from 2008 to 2014. Four rural localities situated along the Padauiri River were the object of this study. Two localities (Bacabal-rio Aracá and the São Sebastião district) were used as controls. LLINs were distributed to all residents of the Padauiri River; assessments were made regarding their use and retention via a semistructured questionnaire, a household register, and direct observation during 5 years.

**Results:**

Overall, 208 individuals participated in the study. In the baseline pilot study (2008), 9.9% of the subjects in the intervention group had slept with mosquito nets the previous night compared with 37.8% of the subjects in the control group. In 2010, this percentage was 43.2% in the intervention group and 50.9% in the control group. Therefore, 1 year after the implementation of the strategy, although there was an increase in the use of mosquito nets in both groups, this increase was significantly higher in the intervention group. This increase in LLINs use did not persist after 5 years of intervention. The households’ evaluation in 2014 showed that 80% of the houses in the intervention group owned at least one LLIN compared with 66% in the control group (p = 0.11); 76% of households in the intervention group owned sufficient LLINs for all family members compared with 50% in the control group (p = 0.007).

**Conclusions:**

High ownership and retention of the LLINs was observed in the intervention group. At 1 year after the distribution of these LLINs, there was a significant increase in their use that was not maintained over the long term. Control strategies must be permanent; however, exploring new strategies is necessary to ensure that the knowledge acquired further modifies the attitudes and behaviours.

## Background

Long-lasting insecticidal nets (LLINs), along with indoor residual spraying (IRS), are the main vector control strategies recommended by the World Health Organization (WHO) for the management of malaria [1, 2]. LLINs are mosquito nets treated with insecticides at the manufacturing unit that do not require any re-impregnation. They are designed to retain their efficacy against mosquito vectors for a minimum of 3 years or 20 standard washes under laboratory conditions [3]. Until 2007, the WHO had directed the distribution of LLINs only to pregnant women, children, and human immunodeficiency virus (HIV)-positive individuals. However, since then, it has been recommended that LLINs should be made available to all individuals at risk in endemic areas, regardless of age—i.e., universal access [4], which is defined as the availability of one mosquito net for every two individuals [5]. The use of LLINs has been shown to be a highly cost-effective strategy for malaria prevention, and it has contributed to a significant reduction in disease morbidity and mortality in recent years [6]. Of the 663 million cases that were circumvented owing to malaria control interventions between 2001 and 2015 in sub-Saharan Africa, it is estimated that 69% were circumvented with the use of LLINs, 21% with artemisinin-based combination therapy, and 10% with IRS [2]. Although studies on LLIN coverage are widespread in the African continent [7–10], studies in Latin America addressing this topic are limited. In the Venezuelan Amazon, the coverage was > 80% of the households owning at least one LLIN at home [11]. Moreover, there has been an increase in the use of LLINs following distribution campaigns in the Latin American [11, 12] and African countries [7, 13].

In Brazil, as a strategy for vector control, the Ministry of Health recommends the use of IRS every 3 months, control of vector mosquito breeding sites and the distribution and installation of LLINs in the residences for free to be used every night, along with awareness activities [14]. The Ministry of Health has officially adopted the use of LLINs since 2011 with the ‘Project on Expansion of Access to Malaria Prevention and Control Measures’, subsidized by the Global Fund to Fight AIDS, Tuberculosis, and Malaria. As part of this programme, 1.1 million LLINs were installed in the houses of priority locations [15].

Although the implementation of this project began in 2009 in Amazonas, with the free distribution of the LLINs in priority locations, data regarding the actual distribution and use of impregnated mosquito nets in this region is scarce. To guarantee the maximum family benefit of this intervention, it is essential to understand the community’s perceptions regarding the use and retention of impregnated nets as well as other factors influencing the individuals whom the programme hopes will sleep safely with the use of these nets [7]. In Brazil, the initial study using impregnated mosquito nets was conducted in Rondônia by Santos et al. [16], wherein a reduction in the anopheline species was observed in the homes with the use of nets. In another study performed in three localities of Rondônia [17], 39.5–55.3% of individuals have been reported to sleep with an LLINs the previous night; moreover, a reduction in malaria cases was observed in two of the localities studied. In a survey conducted in three municipalities of Acre, it was observed that 52% of the individuals slept under an insecticide-treated mosquito net the previous night [18]. The most malarial control intervention evaluations have been conducted a few months, or during the first year, after the implementation. Extensive data on the implementation status of these interventions after 4 or 5 years is lacking. Therefore, the present study aimed to evaluate the use and retention of the LLINs during the first 5 years after the implementation of an integrated strategy for malaria control in the difficult-to-access and high epidemiological risk area of Barcelos, a municipality of the Rio Negro microregion, State of Amazonas, Brazil.

## Methods

This was an interventional study conducted at the community level to examine the effectiveness of an integrated strategy for the control of malaria.

### Area and study population

**Fig. 1:**
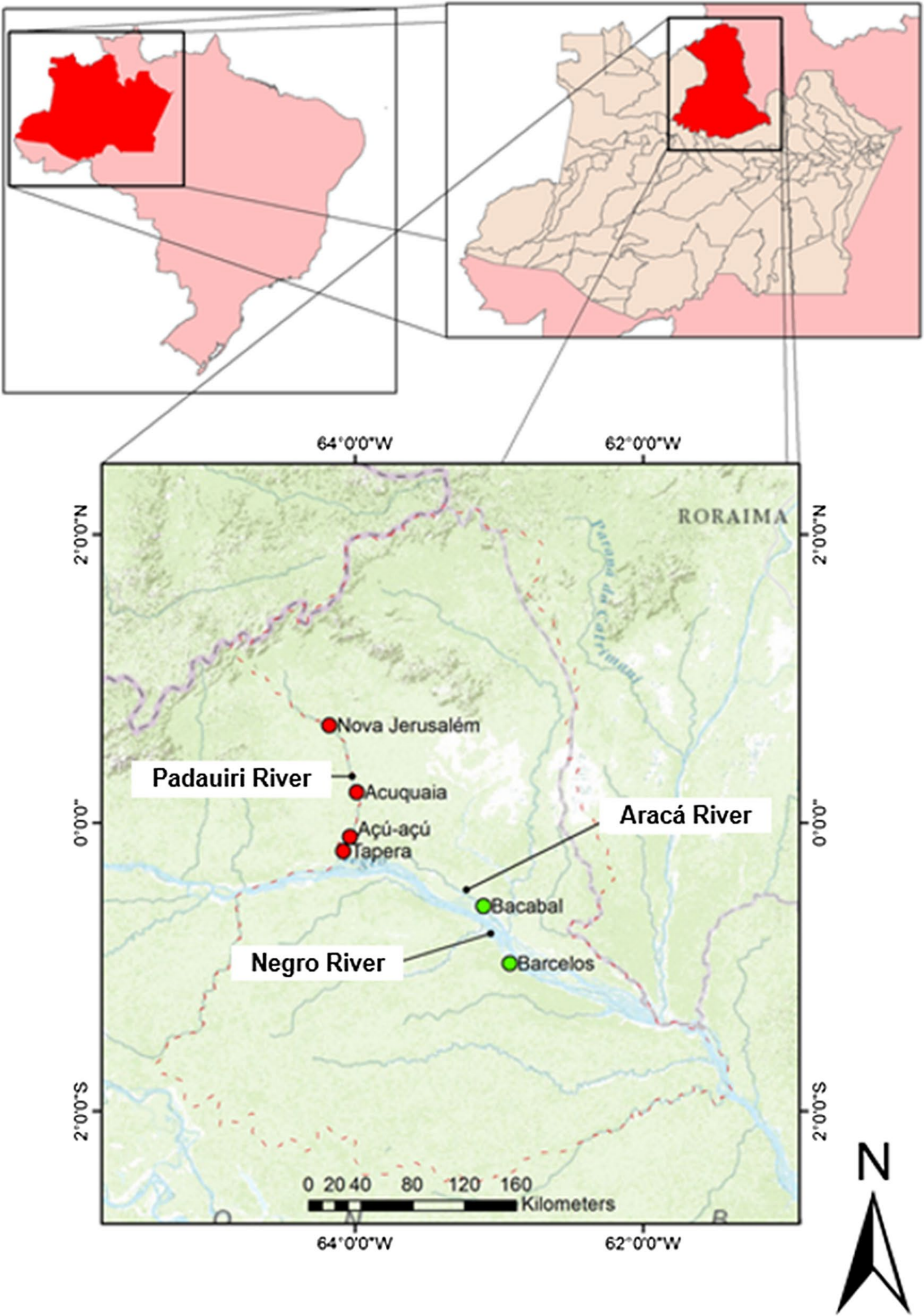
Map of Barcelos showing the study locations. In red: the localities of the intervention group (Padauiri River); in green: the localities of the control group (Bacabal—Rio Aracá and Barcelos, the neighbourhood of São Sebastião—urban area of the municipality)

**Fig. 2:**
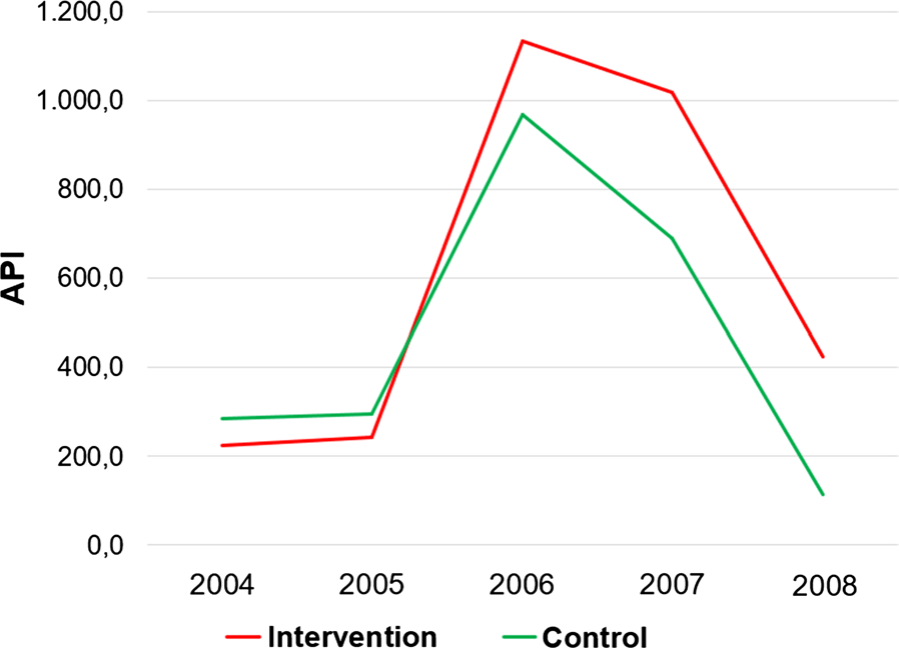
Annual Parasitic Incidence of malaria in intervention and control groups, 2004 to 2008. Source of data: SIVEP/MALÁRIA-MS 2017

The study was conducted in the municipality of Barcelos, an endemic area for malaria in the of Rio Negro microregion, Brazilian Amazon. A total of 3865 malaria cases have been reported from this region in 2016, with an annual parasite incidence (API) of 140.8 cases/1000 inhabitants, characterizing the area as a high epidemiological risk (Fig. 1) [19]. The intervention included all four riverside communities along the Padauiri River, a tributary of the left bank of the Negro River: Tapera, Acú-acú, Acuquaia, and Nova Jerusalém. This area is populated by piaçabeiros (workers who extract fibers from the plant *Leopoldinia piassaba*) who often relocate from one region of the river to another. Further information regarding this population has been published elsewhere [20, 21]. Mean API in the Padauiri River 5 years before the intervention was 473.3 cases/1000 inhabitants (Fig. 2).

The control group comprised the rural locality of Bacabal, on the Aracá River, a tributary of the left bank of the Negro River, with transmission conditions similar to those of the Padauiri River, and the São Sebastião neighbourhood, in the urban area of Barcelos, near Igarapé do Salgado, the largest larval habitat in the urban area of the city (Fig. 1). Mean API in the control region 5 years before the intervention was 400 cases/1000 inhabitants and 472.6 cases/1000 inhabitants in Bacabal and São Sebastião district, respectively (Fig. 2).

### Study design

A baseline pilot study was conducted in 2008, before the commencement of the intervention, with the objective of assessing the knowledge and perceptions regarding malaria and use of mosquito nets among residents. A semistructured questionnaire was applied to all residents in both the intervention and control areas [22].

In 2009, the Ministry of Health-recommended (registry number: 332220031) 0.1% w/w (40 mg/m^2^) deltamethrin-treated LLINs (K-Onet vector control model; Bayer©) were distributed to all four riverside communities along the Padauiri River. Each resident received a bed or hammock mosquito net according to their place for sleeping. In each house, a health worker assembled at least one mosquito net and instructed for a member of the house to assemble one as well. Individual and collective education activities were conducted for ensuring the proper use of mosquito nets, and the relevant cleaning and hygiene instructions were provided.

### Evaluation of the strategy

In 2010, 1 year after the implementation of the project, a partial evaluation of the results was conducted, and new educational activities were undertaken. In June 2014, 5 years after the implementation, another evaluation assessing the use and retention of the LLINs, as well as the effectiveness of the educational strategies, and the reinforcement of the latter with new activities, was conducted. These two evaluations were performed as a part of the integrated strategy for malaria control in this high epidemiological risk area.

### Assessments

Two questionnaires were used for the evaluation: (i) An individual semistructured questionnaire aimed at determining the local residents’ knowledge of malaria and use of mosquito nets, and (ii) a domicile-related questionnaire to be responded only by the head of the family. The main variables of the individual questionnaire included whether the mosquito net was used the previous night, whether the subject shared the mosquito net, whether the mosquito net delivered by the implementation project was still available, and whether the mosquito net was washed. In case the net was washed, data were collected on the number of washes, washing method, and the detergent used. In contrast, the household questionnaire collected data on the number of individuals who had spent the previous night in the house, the number of mosquito nets available in the house, and whether someone had used the mosquito nets the previous night, among other issues. Moreover, direct observation was performed, evaluating variables regarding the use and retention of mosquito nets.

The results of these questionnaires were compared between the intervention and control groups. In addition, when possible, the results were compared with those of the baseline pilot study, using information collected in 2008.

In addition to the two classic indicators of LLIN assessment—the proportion of households owning at least one LLIN and proportion of the population that used a LLIN the previous night, three other indicators were used to assess the universal access and use of LLINs: the proportion of households owning at least one LLIN for every two individuals, proportion of the population with access to a LLIN within their household, and proportion of existing LLINs used the previous night [5]. These five indicators were calculated according to the WHO guidelines [5] (Box 1).

#### Box 1. Calculation of the five indicators of LLIN use based on WHO guidelines (2013)

**Table:** 

Indicator	Calculation based on WHO guidelines (2013)
1) Proportion of households with at least one LLIN	$\frac{{{\text{Number of households surveyed with at least oneLLIN}}}}{{{\text{Total number of households surveyed}}}}*100$
2) Proportion of the population that slept under a LLIN the previous night	$\frac{{{\text{Number of individual who slept under a LLIN the previous night}}}}{{{\text{Total number of individuals responding to the individual questionnaire}}}}*100$
3) Proportion of households with at least one LLIN for every two people	$\frac{{{\text{Number of households with at least one LLIN for every two people}}}}{{{\text{Total number of households surveyed}}}}*100$
4) Proportion of population with access to a LLIN within their household	$\frac{{{\text{Total number of individuals who could sleep under a LLIN if each LLIN in the household is used by two people}}}}{{{\text{Total number of individuals who spent the previous night in surveyed households}}}}*100$
5) Proportion of existing LLIN used the previous night	$\frac{{{\text{Number of LLIN s in surveyed households that were used by anyone the previous night}}}}{{{\text{Total number of LLINs in surveyed households}}}}*100$

The third indicator (i.e., proportion of households owning at least one LLIN for every two individuals) was to be used in conjunction with the first one indicator (i.e., proportion of households owning at least one LLIN) to better define the difference in ownership (i.e., families with/without sufficient numbers of the LLINs). The fourth indicator was intended to define the difference in use (i.e., what part of non-use cannot be explained by the lack of a usable LLIN) [13].

### Statistical analysis

All quantitative variables were accumulated and analyzed using Epi Info version 7.0 (Center for Diseases Control Atlanta—CDC Atlanta, 2014) and GraphPadPrism version 4.00 for Windows (GraphPad Software, San Diego California USA—http://www.graphpad.com). Both univariate and bivariate analyses were performed. Continuous variables were analyzed using the Student *t*-test to compare the means between two data series. Categorical variables were analysed using the Chi square test. In all cases, a p-value of < 0.05 was considered significant.

## Results

### Demographic characteristics

The 2008 baseline pilot study involved 145 participants: 71 (49%) from the intervention group and 74 (51%) from the control group. The first post-intervention evaluation in 2010 involved 136 participants: 81 (59.6%) from the intervention group and 55 (40.4%) from the control group. The final evaluation in 2014, 5 years after the intervention, involved 208 participants: 101 (48.6%) from the intervention group and 107 (51.4%) from the control group. No sex-related differences were observed during the 3 years in the intervention (p = 0.435) and control (p = 0.426) groups; however, in 2014, the proportion of men were higher (56.4%) in the intervention group than in control group (40.2%) (p = 0.019). Median age was 34.5 ± 14.5 years (interval: 15–77) in 2008, 31.3 ± 14.2 years (interval: 14–74) in 2010, and 33.5 ± 12.5 years (interval 16–70) in 2014 in the intervention group and 43 ± 16.2 (interval: 16–82) in 2008, 43.8 ± 15.3 (interval: 19–83) in 2010, and 32.7 ± 16.2 years (interval 15–83) in 2014 in the control group.

Remarkably, no differences were observed between the baseline pilot study and the final evaluation in terms of sex (p = 0.19) and age (p = 0.16). The level of education was lower in the intervention group (uneducated: 6.9% (7/101); < 9 years of schooling: 68.3% (69/101)) than the control group (0.9% (1/107); 33.6% (36/107); p < 0.0000)). Piaçabeiros were only found in the intervention group, wherein they constituted 34.7% of the total participants (35/101); other occupations, such as subsistence agriculture or being a “housewife” were not different in both groups.

### Use of mosquito nets

In the 2008 baseline pilot study, using the indicator “proportion of the population that slept under an LLIN the previous night,” only 9.9% (7/71) of the subjects in the intervention group and 37.8% (28/74) in the control group used a mosquito net the previous night. In the first evaluation in 2010, 1 year after providing the LLINs, these proportions substantially shifted to 43.2% (35/81) in the intervention group and 50.9% (28/55) in the control group. There was a considerable increase in the use of the LLINs in both groups, and this increase was significantly higher in the intervention group (p < 0.001) than in the control group (p = 0.138). In the second evaluation in 2014, 5 years after the implementation of the LLINs, these results were not maintained, and only 14.9% (15/101) of the individuals in the intervention group used a mosquito net the previous night compared with 30.8% (33/107) in the control group (p = 0.006). When comparing the years 2008 and 2014, the use of the mosquito nets in the intervention group increased by 5% (p = 0.03); in contrast, the use decreased by 7% in the control group (p = 0.32), as shown in Table 1. In the baseline study in 2008, the chance of individuals having slept with mosquito nets the previous night in the urban area was 4.3 times higher than that in rural areas (95% CI 1,9–9,5), p = 0.0002; in 2014, this chance was 4.2 times higher (95% CI 2.1–8.4), p < 0.001.

**Table 1:** Use and retention of mosquito nets among study participants. Baseline (2008), 2010 and 2014

	2008					2010					2014					2010–2014
	Intervention		Control		IC 95% p-value	Intervention		Control		IC 95% p-value	Intervention		Control		IC 95% p-value	IC 95% p-value
	N	%	N	%		N	%	N	%		N	%	N	%		
Slept under a mosquito net the night before																
Yes	7/71	9.9	28/74	37.8	*0.18 (0.07–0.45)* *p < 0.01*	35/81	43.2	28/55	50.9	*0.73 (0.37–1.46)* *p > 0.05*	15/101	14.9	33/107	30.8	*0.39 (0.19–0.77)* *p < 0.01*	*4.36 (2.16–8.81)* *p < 0.01*
Other people slept with this same mosquito net																
Yes	5/7	71.4	18/28	64.3	*1.39 (0.23–8.5)* *p > 0.05*	25/35	71.4	24/28	85.7	*0.42 (0.11–1.51)* *p > 0.05*	7/15	46.7	16/33	48.5	*0.93 (0.27–3.15)* *p > 0.05*	*2.86 (0.81–9.99)* *p > 0.05*
How long have slept with mosquito nets																
0–5 years	4/7	57.1	5/28	17.9	*6.13 (1.03–36.45)* *p < 0.05*	33/35	94.3	16/28	57.1	*12.4 (2.47–62.01)* *p < 0.01*	9/15	60	16/33	48.5	1.36 (0.41–4.53) p > 0.05	*12.83 (2.26–72.8)* *p < 0.01*
Likes to sleep with mosquito net																
Yes	53/71	74,6	44/74	59.5	2.01 (0.99-4.1) p > 0.05	69/81	85.2	32/55	58.2	*4.13 (1.83–9.32)* *p < 0.01*	55/101	54.5	49/107	45.8	1.41 (0.89–2.44) p > 0.05	*4.81 (2.32–9.95)* *p < 0.01*
Received mosquito net from project																
Yes	**	**	**	**	**	69/81	85.2	22/55	40	*8.62 (3.81–19.5)* *p < 0.01*	49/101	48.5	2/107	1.9	*49.5 (11.6–211.4)* *p < 0.01*	*6.10 (2.95–12.6)* *p < 0.01*
Still has the mosquito net (retention)																
Yes	**	**	**	**	**	**	**	**	**	**	41/49	83.7	2/2	100	–	**
The mosquito net was hanging																
Yes	**	**	**	**	**	36/69	52.2	19/22	86.4	*0.20 (0.05–0.76)* *p < 0.01*	12/41	29.3	2/2	100	–	*2.63 (1.15–6.0)* *p < 0.05*

– Could not calculate p-value

* Difference between 2010 and 2014 (Intervention)

** There are no data

Individuals who used mosquito nets the previous night were asked whether other individuals used the same LLIN. In the 2010 evaluation, this proportion was 71.4% (25/35) in the intervention group and 85.7% (24/28) in the control group, whereas in the 2014 evaluation, this proportion was 46.7% (7/15) in the intervention group and 48.5% (16/33) in the control group. In terms of sharing the LLINs, 42.9% (3/7) of the participants in the interventional group and 37.5% (6/16) of those in the control group shared the LLIN with one individual; further, 57.1% (4/7) of those in the interventional group and 62.5% (10/16) of those in the control group shared the LLINs with two or three individuals (Table 1).

Regarding the duration of use, most participants in the interventional group used LLINs for ≤ 5 years in all the surveyed years (2008, 2010, and 2014), whereas most participants in the control group used the LLINs for > 10 years in the 2008 survey and for the last 5 years in the 2010 and 2014 surveys. When being queried whether the individual prefers or would prefer to sleep with mosquito nets, in 2008 74.6% (53/71) of participants in the intervention group and 59.5% (44/74) of those in the control responded affirmatively. However, in 2010, this proportion was 85.2% (69/81) in the intervention group and 58.2% (32/55) in the control group, and in 2014, it was 54.5% (55/101) in the intervention group and 45.8% (49/107) in the control group. Some of the reasons expressed for preferring the nets were as follows: “to avoid insects/bugs/mosquitoes,” “to prevent carapanã (popular name used in the study area for the anopheline vector),” “to sleep well and protected from insect interference,” and “to avoid malaria.” Some of the reasons for not preferring the mosquito nets were as follows: “it is very hot,” “not accustomed,” “feel stifled,” and “it is distressing and uncomfortable” (Table 1).

In 2010, 85.2% (69/81) of the participants in the intervention group and 40% (22/55) of those in the control group indicated that they had received mosquito netting from the project. In 2014, 48.5% (49/101) of the intervention group and 1.9% (2/107) of the control group indicated the same, whereas 83.7% (41/49) of the intervention group expressed that they still had it (Table 1). Of those who did not have the LLIN at that point, 25% (2/8) responded that they had donated the net to someone, 12.5% (1/8) reported having loaned it out, and 62.5% (5/8) revealed that the mosquito net had torn and was thrown away (Table 1).

It was observed that 52.2% (36/69) of the participants in the intervention group in 2010 and 29.3% (12/41) in 2014 were using the nets (which were generally observed hanging on the walls of the house) (Table 1).

In 2014, entirely 100% of these individuals reported that sleeping with mosquito nets prevented them from contracting malaria and prevented mosquitoes from biting them while sleeping. Regarding the adverse effects from the nets, 57.1% (28/49) of the individuals in the intervention group reported experiencing some symptoms when they started using the mosquito nets. Among those cited were “blazing” and “itching.” The duration of such symptoms ranged from < 1 h to 2 months. In contrast, only 2.4% (1/41) of the individuals in the intervention group reported currently experiencing any symptoms when using the mosquito net.

### Maintenance and physical condition of mosquito nets

Of the individuals who received the LLINs, at the end of the evaluation in 2014, 93.9% (46/49) had washed it; of these, 63% (29/46) reported washing it 1–5 times, 95.7% (44/46) used cold water, 60.9% (28/46) used detergent or soap powder, and 28.3% (13/46) used sanitary water. It was observed that 47.8% (22/46) of the individuals dried the LLINs in the shade. Direct observation showed that 95.1% (39/41) of the existing LLINs appeared to be clean. The presence of holes was observed in 53.7% (22/41) of the nets, with 31.8% (7/22) containing 1–5 holes and 59.1% (13/22) containing 6–10 holes. Of these, 59.1% (13/22) exhibited holes of approximately 1–3 cm. Some mosquito nets, in addition to the presence of holes, had tears (Table 2).

**Table 2:** Maintenance and physical condition of mosquito nets year 2014

	Intervention	
	N	%
Received the project mosquito net		
The mosquito net was washed	46/49	93.9
How many times has it been washed?		
1–5 times	29/46	63
6–10 times	5/46	10.9
More than 10 times	2/46	4.4
Not know	10/46	21.7
Washed the mosquito net with cold water	44/46	95.7
Used to wash the mosquito net		
Detergent/soap powder	28/46	60.9
Bar soap	11/46	23.9
Bath soap	4/46	8.7
Not know	2/46	4.4
Does not reply	1/46	2.2
Used bleach water		
Yes	13/46	28.3
How did the mosquito net dry?		
Sun	23/46	50
Shadow	22/46	47.8
Still owned the project mosquito net		
Physical condition of the mosquito net	39/41	95.1
Clean		
Dirty	1/41	2,4
The mosquito net has holes	22/41	53.7
Number of holes	7/22	31.8
1–5 holes		
6–10 holes	13/22	59.1
More than 10 holes	1/22	4.6
Average holes size		
1–3 cm	13/22	59.1
4–6 cm	6/22	27.3
More than 6 cm	2/22	9.1

### Indicators of the use of mosquito nets

**Fig. 3:**
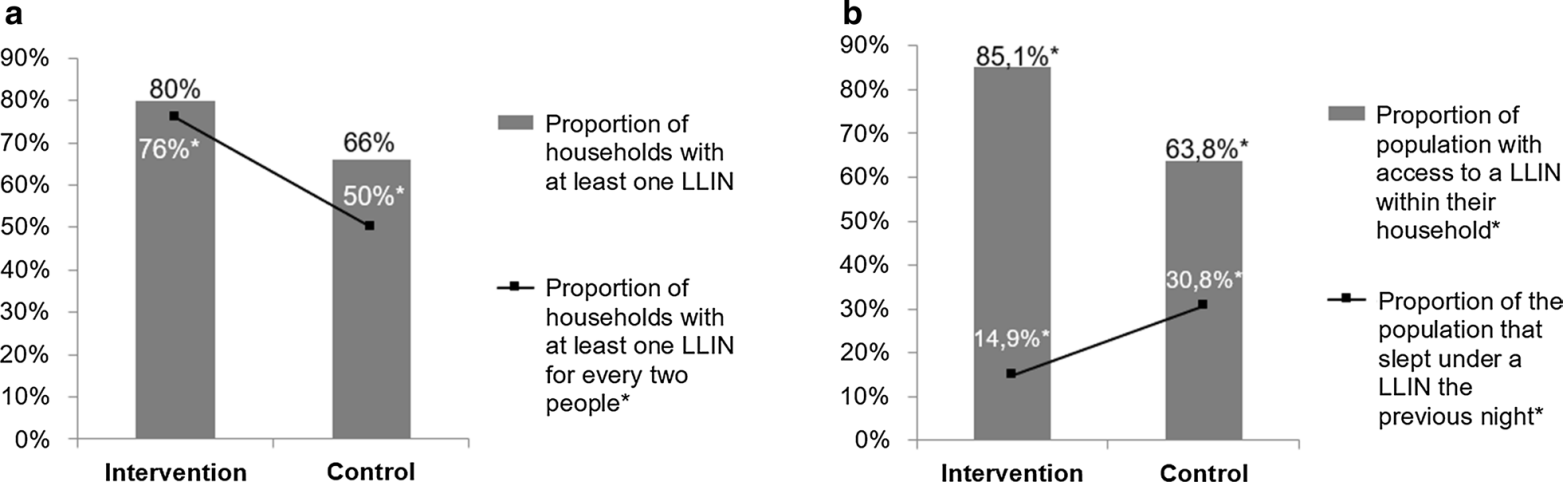
Indicators of use of mosquito nets. **a** Proportion of households with at least 1 LLIN/proportion of households with at least 1 LLIN for every 2 people: 2014. *p-value < 0.05; **b** Proportion of population with access to a LLIN within their household/proportion of population that slept under a LLIN the previous night: 2014. *p-value < 0.05

Overall, 100 families participated in the present study, of which 50% (50/100) belonged to the intervention group and the remaining belonged to the control group. The mean number of inhabitants per family in the intervention group was 4 ± 2.2 (minimum of 1 and maximum of 11) persons. In the control group, the mean number of inhabitants per family was 5.6 ± 2.8 (minimum of 1 and maximum of 14). On assessing the number of individuals who slept the previous night in the house, the mean in the intervention group was 3.5 ± 1.8 (minimum of 1 and maximum of 7) individuals per family. In the control group, the mean was 4.5 ± 3.0 (minimum of 0 and maximum of 14) individuals per family. It was found that 80% (40/50) of the families in the intervention group and 66% (33/50) of those in the control group owned at least one mosquito net (p = 0.11). In practice, 76% (38/50) of the families in intervention group and 50% (25/50) of those in the control group owned sufficient nets for all inhabitants (p = 0.01), with the understanding that one mosquito net can be used by up to two individuals (Fig. 3; Box 1). Moreover, 27.5% (11/40) of the families in the intervention group and 3% (1/33) of those in the control group reported that they received their mosquito nets via the project, whereas 65% (26/40) of those in the intervention group and 81.8% (27/33) of those in the control group received their LLINs from the state, and 2.5% (1/40) of those in the intervention group and 12.1% (4/33) of those in the control population purchased it.

On assessing the access to the LLINs, 85% (148/174) of the intervention group and 64% (143/224) of the control group was shown to have access to an LLIN (p = 0.00); 14.9% (15/101) of the intervention group and 30.8% (33/107) of the control slept with mosquito nets the previous night (p = 0.01), as shown in Fig. 3. In the intervention and control groups, respectively, 13.5% (23/171) and 57% (69/121) of the existing bed nets were used by someone the night before the survey (Table 3).

**Table 3:** Indicators of use of mosquito nets year 2014

	Intervention		Control		Total		p-value
	N	%	N	%	N	%	
Number of households	50	50	50	50	100	100	–
Proportion of households with at least one LLIN *	40	80	33	66	73	73	0.11
Proportion of households with at least one LLIN for every two people	38	76	25	50	63	63	0.01
Number of persons responding to the individual questionnaire	101	48.6	107	51.4	208	100	–
Proportion of the population that slept under an LLIN the previous night	15	14.9	33	30.8	48	23.1	0.01
Number of people who spent the previous night in the house	174	43.7	224	56.3	398	100	–
Proportion of population with access to an LLIN within their household	148	85.1	143	63.8	291	73.1	0.00
Total number of LLINs in households surveyed	171	58.6	121	41.4	292	100	–
Proportion of existing LLINs used the previous night	23	13.5	69	57	92	31.5	0.00

## Discussion

The strategy of distributing mosquito nets impregnated with insecticides has been critical in the reduction of malaria worldwide and is one of the main interventions toward the goal of elimination of the disease [2]. The present study followed the implementation of this strategy in an area of difficult geographical access, wherein mosquito nets were distributed to 100% of the residents in an intervention area. The evaluation of the use and retention of the nets were undertaken 1 and 5 years after the intervention, which was in contrast to most studies of this type wherein the evaluations usually occur only within the first 12 months after the distribution of the nets [7, 11, 13]. At 12 months after implementation of the strategy, in the intervention area, the coverage of 85.2% of the households and sufficient LLINs for all family members were observed. This coverage was slightly lower than that found by Alvarado et al. [11] in the Venezuelan Amazon, wherein the proportion of households with at least one mosquito net was 93.7%. A study conducted in Nigeria has shown a good coverage of households with at least one LLIN (74.5%), but the number of households owning sufficient LLINs for all family members was substantially low (27.2%) [13]. After 5 years of intervention, the coverage of households owning at least one LLIN in the intervention group was 80%. Despite the fact that universal coverage (100%) was expected, it is easy to understand that there was an increase in the number of inhabitants in these localities, either by the birth of children or by the mobility of individuals who migrated to work of extract fibers from the plant *Leopoldinia piassaba*. This evolution was observed during the interviews conducted in 2014. However, in the intervention area, the coverage of mosquito nets, as well as owning sufficient LLINs for all members of the family, was greater than that in the control area, showing a positive result of the strategy of using mosquito nets. According to the WHO, in sub-Saharan Africa, the ownership of at least one LLIN in the family increased from 50% in 2010 to 80% in 2016. However, only 43% owned sufficient bed nets for all family members by that year [23]. Similar findings were observed in southern Ethiopia [9] and in the Democratic Republic of Congo [10], wherein although a coverage of 80%–90% of households owning at least one mosquito net was found, sufficient LLINs for all family members were not available. In a study performed in a region of eastern Ethiopia, a coverage of just over half the households (57.9%) was found, although 68% of the inhabitants in this country live in malaria risk areas [24].

### Use of the LLINs

The proportion of individuals who used mosquito net the previous night increased 1 year after the distribution of the LLINs in the intervention area; however, this was not maintained over time and decreased after 5 years. In all three evaluations, the proportion of individuals using the LLINs was higher in the control group than in the intervention group. This result could be explained because a majority of the inhabitants of the control group resided in the urban area wherein there is a greater variety and quantity of mosquitoes and more access to the LLINs. Furthermore, the use of the LLINs the urban area was at least four times greater than that the rural areas, in both the baseline pilot study and in 2014. In rural areas, individuals tend to use mosquito nets only when there are significant mosquitoes and malaria cases.

Moreover, it was observed that in the intervention group, after 5 years of strategy implementation, only 29.3% of the nets were hanging in the house. In this area, malaria is a seasonal disease with the highest number of cases occurring at the end of the rainy season when there is a greater presence of anophelines. Anopheles darlingi has a endophilic behaviour and is the main vector of malaria in the Amazon region. The anophelines exhibit a peak of activity in the evening and morning twilight and continued their activity throughout the night in this region [25]. In Costa Marques, State of Rondonia, Brazil, a decreased number of anophelines collected intra domiciliary was observed [16]. This result could reflect the repellent action of the LLINs. Although one of the main reasons associated with the residents preferring to sleep with mosquito nets in both the intervention and control groups is prevention against being bitten by anophelines, unfortunately, this is not reflected via a continued use of the LLINs. Further efforts are required to increase the perception of protection that mosquito nets can provide to individuals in endemic areas to create a “mosquito net culture,” despite variations in mosquito densities throughout the year, as Koenker et al. [17] have shown.

Another interesting finding was observed among individuals in the intervention group who slept with mosquito nets the previous night. In 2008, 71.4% shared the mosquito net with another family member; however, in 2014 this proportion had decreased to only 46.7% (p = 0.0009). It is possible that individuals are less frequently sharing mosquito nets because they currently have more access to them, with control programmes continuing to distribute the LLINs in this area. The greatest improvements in the use of mosquito nets have been observed following massive community distributions [8]. Despite the decrease in the proportion of sharing, 57.1% of the individuals shared with two others; i.e., three people slept under the same LLIN, despite it being recommended that no more than two individuals should share the same net [5].

On comparing access to the nets with actual use, it was observed that although 85.1% of the individuals in the intervention group had access to an LLIN in 2014, only a remarkable 14.9% used one the previous night. Moreover, a similar relationship occurred among the individuals in control group, wherein although 63.8% had access, only 30.8% used them the previous night. These data demonstrate that despite the access to the LLINs in the intervention area was higher than in the control area, the gap between access and use was also higher in this group, showing that the lack of a mosquito net was not the reason for the net not being used the previous night. The reason appears to be cultural or psychologically oriented. In both cases, the estimate of use was less than the ownership estimate, suggesting a significant difference between owning and using [11]. Some studies that specifically evaluated the use of mosquito nets have found that between 15 and 50% of distributed LLINs remain unused [26–28]. Therefore, ownership is not the sole obstacle to achieving reductions in malaria morbidity and mortality associated with the use of the LLINs. Individuals, who own mosquito nets [or to whom the nets are available], should use them to have an impact on malaria reduction [29]. The results differ from those of Kilian et al. [13], wherein the proportion of the population that used a LLIN the previous night was 41.3%—only slightly lower than the access rate (50%)—indicating a high general level of use among those who have access. However, these studies do not include data on vector behaviour in the targeted areas, thereby inhibiting an analysis of this aspect of the issue.

In 2016 in sub-Saharan Africa, 54% of the at-risk population slept under an LLIN, which is a substantial increase from 30% in 2010 [23]. It has already been demonstrated that LLINs are important for protecting all individuals in a community, including those who do not sleep under a mosquito net [11]. Such a community effect from the LLINs is attributable to the fact that insecticides incorporated in their mesh kill the vectors, reducing their overall density in the community. In addition, it is known that the LLINs can prevent only up to 54% of malaria cases in a given area because their action occurs predominantly when people are inside the mosquito nets while sleeping. This happens in a variable way according to the age and behaviour of the residents [12], but the coverage seems to be an important factor.

The main reason reported by locals for not using mosquito nets is the heat. This finding is similar to that of the study by Cohee et al. [7] conducted in Uganda and that conducted in Bukoba and Zanzibar [30], wherein participants said they felt crammed, uncomfortable, hot, and itchy when they slept under a mosquito net. In a study conducted in eastern Ethiopia [24], the main reason reported for not using a mosquito net (69.9%) was “because there is no mosquito in the area.” In a study by Egrot et al. [31] in southern Benin that interviewed 91 individuals, 56 mentioned that a possible cause for the non-use of the LLINs is that they can ignite and cause serious material damages and bodily injuries or even death. Of these individuals, 34 narrated specific events that they heard or experienced, where fire was always related to the internal use of a lantern or candle that accidentally came into contact with a mosquito net. The review by Pulford et al. [29] has shown that the main reason for not using mosquito nets were discomfort, chiefly due to the heat, and the low density of mosquitoes. These authors have expressed that if a motive to use a mosquito net is the density of the mosquitoes, it seems apparent that in areas where this density falls as a result of increased LLINs coverage, indoor spraying, or by other measures, the motivation to use the nets may decrease. It may be possible to achieve greater use of nets among this population via behaviour-modifying education strategies. Regarding the personal discomfort, modifications to the mosquito nets to make them more comfortable would likely complement any educational campaign in a useful way.

### Retention of the LLINs

Five years after the distribution of the LLINs, retention was high in the intervention group (83.7%). Similar results were found by Cohee et al. [7]. A few individuals in the control group owned the LLINs delivered during the campaign probably because they relocated from the intervention area to live in one of the control areas. All participants in that study said sleeping with mosquito nets prevented them from contracting malaria and from mosquitoes biting them while sleeping without net. More broadly, in the study by Cohee et al. [7], 80% of the participants agreed that the LLINs are used to prevent malaria, and in another investigation conducted in Ethiopia, this proportion was 97.6% [32]. Despite the existence of knowledge regarding the importance of mosquito nets to prevent malaria, this perception is not producing a change of behaviour, at least in the long term.

Concerning the maintenance of the nets, 93.9% were washed 1–5 times in the intervention area. The LLINs, under field conditions, have a duration of 3 years, depending on the form and frequency of washing, because their biological effectiveness without a new treatment is retained for at least 20 washes under laboratory conditions [3]. Therefore, the inhabitants of the study area are following the standards of care for the LLINs, without exceeding the recommended washing frequency. Most (60.9%) used soap powder or detergent compared with only 23.9% that used the recommended bar soap. It should be noted that the LLINs were delivered following an educational strategy that was apparently not sufficient to produce adequate mosquito net washing practices. A similar result was found by Tomass et al. [9], wherein 44.2% of the respondents expressed that they dried the LLINs in the sun. According to the Centers for Disease Control and Prevention (CDC) [33], the insecticide pyrethroid does not decompose rapidly unless exposed to sunlight. The CDC instructs that the nets should be washed with neutral soap and cold water and dried in the shade for better conservation of the insecticide [34]. Clearly, it was observed that this population was not properly washing the mosquito nets and that this may be harming the effectiveness of the insecticide. Studies to evaluate the retention of insecticides under field conditions are required.

Regarding the physical condition of the nets in the intervention group, it was observed that 5 years after the LLINs distribution, although most of the nets were clean, more than half contained holes and some were torn. In the study by Cohee et al. [7], 4 out of 32 mosquito nets were found torn and 3 of these were still assembled. In Ethiopia, only 10.3% of the LLINs contained holes that could allow mosquitoes to enter [32]. Studies similar to this one, comparing the state of the LLINs after a long period of use could not be found. However, it has already been observed that in the impregnated mosquito nets, the irritant effect of the insecticide causes repellency, thereby decreasing the survival or changing the behaviour of mosquitoes coming into contact with the insecticide. Therefore, the repellent effect of the impregnated mosquito net would exert its protective action, despite being damaged and having tears, thereby continuing to reduce the possibility of infective bites [35].

Regarding possible adverse effects with the use of the nets, 57.1% of the individuals in the intervention group reported experiencing some symptom when they started to use the nets. The main symptoms cited were “blazing” and “itching.” These may be due to the insecticide contained in the mesh of the mosquito net, which in some individuals caused an allergic reaction or irritation. The results of this study differ from those found by Alvarado et al. [11], wherein a substantially low percentage (0.4%) of users reported mild discomfort that spontaneously disappeared after the initial days of use. In this study, after 5 years, only 2.4% of the individuals reported experiencing some symptoms. These individuals no longer experience these symptoms, probably because the mosquito nets have already lost part of the insecticide.

Finally, the results show that despite individuals having access to the LLINs, new strategies are necessary to increase long-term use. In numerous localities, there is a need for permanent education measures to ensure that individuals do not lose the practice of using the mosquito nets. Moreover, the need to develop communication strategies for behaviour change was observed in the studies with African populations [10, 32]. Assessing integrated disease control strategies in endemic areas is difficult. Some studies have used mathematical models to measure how far the effect of an intervention is due to the use of a new technology or product of the synergistic action of various strategies [36]. In the present study, there was no attempt to measure the impact of the use of nets for the reduction of malaria, but only the behaviour of individuals regarding the use and retention of the LLINs.

## Conclusions

The data of this study demonstrated that 5 years after the intervention, there was a high ownership and retention of the LLINs among the individuals who received these nets from the project in 2009. However, using as an indicator, the proportion of individuals sleeping under a mosquito net the previous night, 1 year after the distribution of bed nets, a significant increase was observed in the use of mosquito nets that was not maintained over the long term, suggesting that either this particular indicator is not a good indicator of use or that the programme failed to change behaviours over the longer term, particularly in rural hypoendemic areas with the seasonal transmission of malaria. However, despite a 5% increase in the use of the LLINs in the intervention group over 5 years, in the control group, there was a decrease of 7% in the use of these nets. Therefore, it is necessary to search for other more sensitive indicators of use and more persuasive educational programmes. The results suggest a significant difference between owning and using. The authors conclude that the strategies used must be permanent in areas of high epidemiological risk and difficult geographical access, where people live at low socio educational levels and that it is necessary to search for new interventions to ensure that the knowledge acquired results in a permanent modification of attitudes and behaviours.

## Abbreviations

API: annual parasite incidence
CDC: Centers for Disease Control and Prevention
IRS: indoor residual spraying
LLINs: long-lasting insecticidal nets
WHO: World Health Organization
