# Correction to: Use and retention of long-lasting insecticidal nets (LLINs) in a malaria risk area in the Brazilian Amazon: a 5-year follow-up intervention

**DOI:** 10.1186/s12936-019-2761-7

**Published:** 2019-04-09

**Authors:** Jessica de Oliveira Sousa, Bernardino Claudio de Albuquerque, José Rodrigues Coura, Martha Cecilia Suárez-Mutis

**Affiliations:** 1 Laboratory of Parasitic Diseases, Institute Oswaldo Cruz/Fiocruz, Av Brasil 4365. Pavilhão Artur Neiva, Rio de Janeiro, RJ CEP: 21040-900 Brazil; 2 Foundation of Health Surveillance of Amazonas, Av. Torquato Tapajós, 4.010, Colônia Santo Antônio, Manaus, AM CEP 69.093-018 Brazil

## Correction to: Malar J (2019) 18:100 10.1186/s12936-019-2735-9

Following publication of the original article [1], the corresponding author flagged that the particle ‘de’ in their name had been placed incorrectly.

The particle had been positioned as: **Jessica Oliveira de Sousa.**

However, the correct positioning is: **Jessica de Oliveira Sousa.**

As such, the name has now been corrected accordingly in the published article.

The publisher apologizes for this processing error.
